# Protein-lipid interactions and protein anchoring modulate the modes of association of the globular domain of the Prion protein and Doppel protein to model membrane patches

**DOI:** 10.3389/fbinf.2023.1321287

**Published:** 2024-01-05

**Authors:** Patricia Soto, Davis T. Thalhuber, Frank Luceri, Jamie Janos, Mason R. Borgman, Noah M. Greenwood, Sofia Acosta, Hunter Stoffel

**Affiliations:** ^1^ Department of Physics, Creighton University, Omaha, NE, United States; ^2^ Omaha Central High School, Omaha, NE, United States; ^3^ Department of Chemistry and Biochemistry, Creighton University, Omaha, NE, United States; ^4^ Omaha North High School, Omaha, NE, United States

**Keywords:** Prion protein (PrP), Doppel protein, molecular dynamics simulations, protein-lipid interactions, peripheral membrane protein binding

## Abstract

The Prion protein is the molecular hallmark of the incurable prion diseases affecting mammals, including humans. The protein-only hypothesis states that the misfolding, accumulation, and deposition of the Prion protein play a critical role in toxicity. The cellular Prion protein (PrP^C^) anchors to the extracellular leaflet of the plasma membrane and prefers cholesterol- and sphingomyelin-rich membrane domains. Conformational Prion protein conversion into the pathological isoform happens on the cell surface. *In vitro* and *in vivo* experiments indicate that Prion protein misfolding, aggregation, and toxicity are sensitive to the lipid composition of plasma membranes and vesicles. A picture of the underlying biophysical driving forces that explain the effect of Prion protein - lipid interactions in physiological conditions is needed to develop a structural model of Prion protein conformational conversion. To this end, we use molecular dynamics simulations that mimic the interactions between the globular domain of PrP^C^ anchored to model membrane patches. In addition, we also simulate the Doppel protein anchored to such membrane patches. The Doppel protein is the closest in the phylogenetic tree to PrP^C^, localizes in an extracellular milieu similar to that of PrP^C^, and exhibits a similar topology to PrP^C^ even if the amino acid sequence is only 25% identical. Our simulations show that specific protein-lipid interactions and conformational constraints imposed by GPI anchoring together favor specific binding sites in globular PrP^C^ but not in Doppel. Interestingly, the binding sites we found in PrP^C^ correspond to prion protein loops, which are critical in aggregation and prion disease transmission barrier (β2-α2 loop) and in initial spontaneous misfolding (α2-α3 loop). We also found that the membrane re-arranges locally to accommodate protein residues inserted in the membrane surface as a response to protein binding.

## Introduction

**FIGURE 1:**
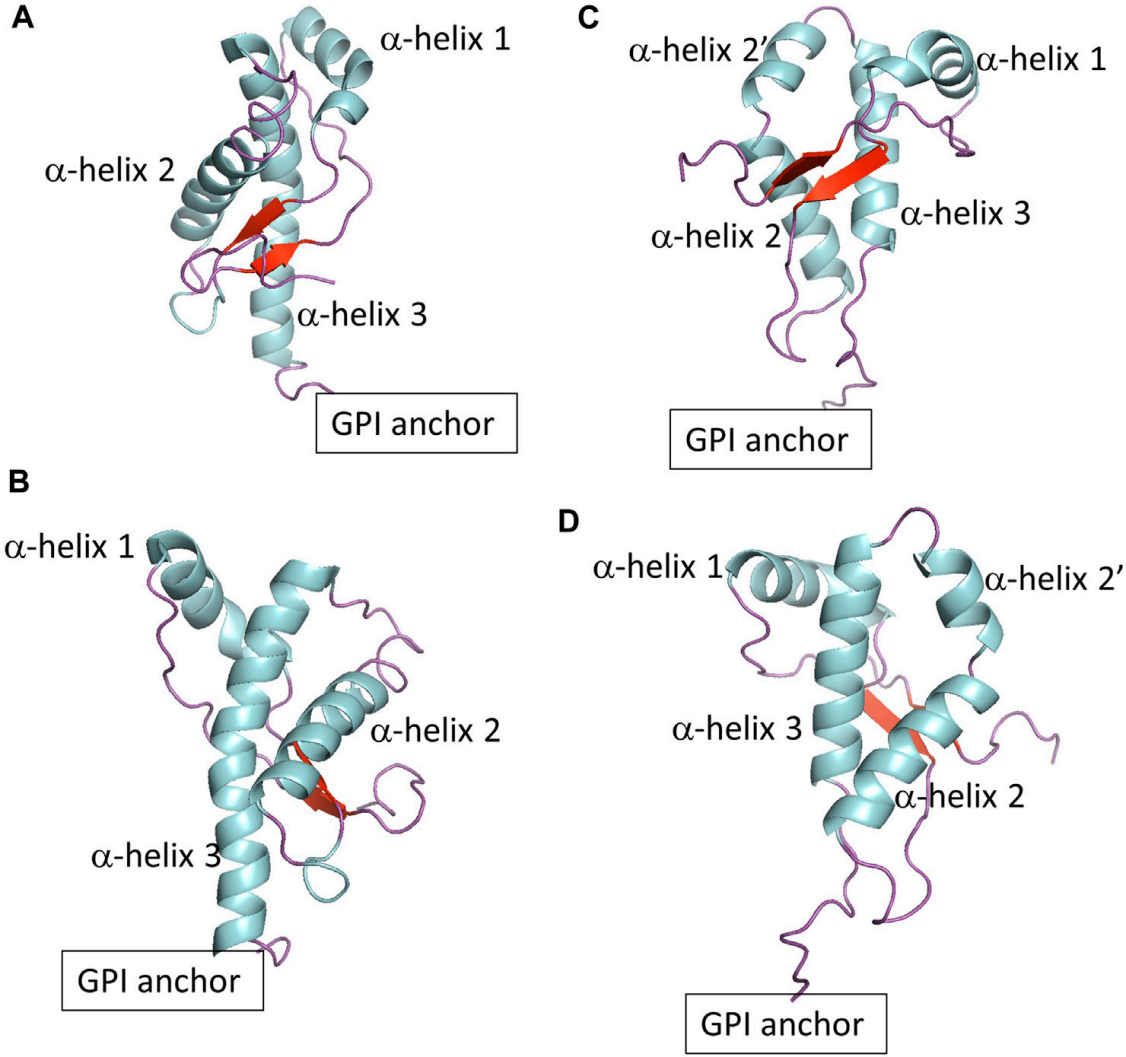
**(A,B)** Show two different views of globular PrP^C^. **(C,D)** Show two different views of globular Doppel protein.

Prion protein behavior represents a novel paradigm. Prion proteins propagate biological information by templated conversion of the cellular conformer of the host-encoded Prion protein (PrP^C^) to the infectious misfolded scrapie conformation (PrPSc) in the absence of specific nucleic acids (Alper et al., 1966; Deleault et al., 2007). PrPSc is the main component of prions, infectious agents responsible for transmissible spongiform encephalopathies (TSE) (Griffith, 1967; Prusiner, 1982), also known as prion diseases. The disorders are inevitably fatal neurodegenerative diseases in mammals, such as Creutzfeldt-Jakob disease in humans and Chronic Wasting Disease in cervids. The Prion protein is a glycoprotein anchored to the extracellular leaflet of the plasma membrane via a glycosylphosphatidylinositol (GPI) molecule. The mature Prion protein consists of two domains with a similar number of residues: An intrinsically disordered domain that includes an octarepeat region and a polybasic region, both flanked by hydrophobic fragments. The globular domain (termed globular PrP^C^ in this article) contains three α−helices and two β−sheets, with a disulfide bond that connects the second and third helices (Figures 1A, B). The GPI anchor tethers the Prion protein to cholesterol and sphingomyelin-rich domains via the C-terminus of the globular domain. The physiological function of the Prion protein is not fully established, although the cellular location hints at roles in cell signaling, myelination, and ion metabolism (Watts et al., 2018; Alves Conceição et al., 2023).

Although evidence suggests that Prion protein misfolding occurs on the surface of the plasma membrane, a structural model of protein conformational conversion is elusive. Prion protein conversion correlates inversely with sphingomyelin levels in the plasma membrane (Naslavsky et al., 1999). Biochemical characterization and enrichment methods showed that different phospholipids scaffold Prion protein conversion and propagation. However, the level of toxicity of the resulting aggregates was different for each lipid. Conversion in the presence of the anionic phospholipid POPG results in *bona fide* prions (Wang et al., 2010; Srivastava, S. and Baskakov, 2015). In contrast, conversion in the presence of phosphatidylethanolamine (PE), a zwitterionic phospholipid, produces prions with a low toxic load. Experiments using rat brain homogenates suggest a relative preference of the Prion protein for phosphocholine, PC, and cholesterol molecules (Brügger et al., 2004). Microarray analysis points to the critical role of cholesterol in prion pathophysiology (Bach et al., 2009). Evidence from experiments (Fantini et al., 2004; Caughey et al., 2009) suggests that a likely pathway for the initiation of the Prion protein conversion is facilitated by the coalescence of cholesterol domains, one with the cellular form of the Prion protein attached to it, the other with the β−sheet rich form.

The anchoring state of the Prion protein may influence Prion protein conformational conversion. Evidence suggests the dissociation between infectivity (i.e., self-replication) and neurotoxicity is modulated by the anchoring state of the Prion protein (Chesebro et al., 2005). Likely, the GPI-anchor constrains the conformational space of the Prion protein toward a toxic state (Noble et al., 2015). An alternative explanation (Aguilar-Calvo et al., 2017) suggests that anchor and anchorless prions represent each different disease phenotype.

To develop a conceptual framework that fully depicts Prion protein conformational conversion and propagation, a baseline of how mature PrP^C^ interacts with the plasma membrane is needed. The heterogeneity of the molecular environment, including competing energetics of inter-molecular interactions happening in a wide range of time-scales, challenges experimental and computational studies. To identify the driving forces underlying PrP^C^–membrane interactions, we monitored coarse-grained molecular dynamics simulations that mimic the conformational behavior of the globular domain of the anchored Prion protein to model membrane patches.

To explore the role of protein sequence on protein-lipid interactions, we also included the Doppel protein, a member of the mammalian prion glycoprotein family (Westaway et al., 2011). The Doppel protein is GPI anchored to the cell surface and, in contrast to PrP^C^, is expressed mainly in the male reproductive tract and not in central nervous system tissue. There is no evidence of a relationship between misfolding of the Doppel protein and prion disease pathobiology. The Doppel protein sequence is, on average, only 25% similar to PrP^C^. However, the topology of the globular domain of Doppel resembles that of globular PrP^C^ except at the fragment between α−helix 2 and α−helix 3 (Figures 1C, D). While PrP^C^ shows a threonine-rich loop, Doppel shows a short α−helix. In addition, the Doppel protein contains an additional disulfide bond connecting the β2−α2 loop to the flexible C-terminus of the globular domain. Because PrP^C^ and Doppel are GPI anchored to cholesterol and sphingomyelin-rich membrane domains, the two proteins may be exposed to similar molecular environments, perhaps interact with each other or have common interacting proteins (Watts et al., 2009; Westaway et al., 2011). Molecular modeling studies indicate that while PrP^C^ exhibits higher thermal stability, native Doppel is more stable due to higher free energy barriers against non-native conformations (Baillod et al., 2013).

To investigate the modes of association between PrP^C^ and Doppel proteins with a model cell membrane, we conducted simulations of the globular domain of each protein in the unglycosylated form on three distinct membrane patches:

• The first patch, the *SM patch*, models a sphingomyelin and cholesterol-rich membrane domain (30% POSM, 40% POPC, 30% cholesterol) where the GPI-anchored PrP^C^ and Doppel protein are likely to be situated.
• The second patch, the *PC patch*, consists of a binary mixture of POPC and cholesterol (70% POPC, 30% cholesterol); and simulates an only cholesterol-enriched domain.
• The third patch, the *PG patch*, examines the impact of a negatively charged head group, POPG (30% POPG, 40% POPC, 30% cholesterol), on the protein-membrane modes of association.

The simulations allow us to interrogate how protein and membrane surface respond to each other, shedding light on the driving forces of association. Here, we show that globular PrP^C^ associates with the membrane surface via loops flanking α−helix 2 regardless of the lipid composition of the membrane. Binding sites that include protein residues with an OH group in the side chain, and not amidic residues, elicit remodeling of the membrane surface, measurable in the time scale of our simulations. For the Doppel protein, we observed binding events only when the protein was simulated in the PG patch. And, the response of the membrane surface was non-measurable in our simulations, consistent with most residues involved in the binding being of amidic nature. Our findings demonstrate that specific protein-lipid interactions and conformational constraints imposed by GPI anchoring together favor binding sites in globular PrP^C^ but not in Doppel, and that the membrane re-arranges locally to accommodate protein residues that insert in the membrane surface.

## Methods

The initial conformation of the mouse PrP^C^ protein structure (pdb id 2L39) and the mouse Doppel protein structure (pdb id 1I17) were obtained from the solution NMR structure deposited in the protein data bank. For each protein, the martinize.py (de Jong et al., 2013) script was used to coarse-grain the protein structure and generate the topology files using the Martini 2.2P forcefield (Monticelli et al., 2008; de Jong et al., 2013). The ElNeDyn elastic network model was used to maintain the tertiary structure of the protein, with a 500 kJ mol^−1^ nm^−2^ force constant; the lower and upper elastic bond cut-offs were set to 0.5 and 0.9 nm, respectively. The same GPI anchor molecule was used for both proteins. The coarse-grained model was kindly developed by César A. López following the Martini forcefield principles for glycolipids (López et al., 2013) and based on an all-atom model previously used to model the initial steps of Prion protein conversion (DeMarco and Daggett, 2009; Wu et al., 2015).

The model membranes were generated using CharmmGUI (Jo et al., 2008; Brooks et al., 2009; Lee et al., 2016) and the Martini forcefield (Marrink et al., 2004) for the following lipids: palmitoyloleoyl phosphatidylcholine (POPC), phosphatidylglycerol (POPG), sphingomyelin (POSM), and cholesterol. We generated three model membrane patches: POSM:POPC:cholesterol, POPC:cholesterol, and POPG:POPC:cholesterol. The ratio of lipids of the ternary mixtures was set to 30:40:30 and the ratio of the binary mixture was 70:30. The GPI-anchored protein and membrane patch were solvated in a water box with the PW water model (Yesylevskyy et al., 2010). Counterions (Na^+^ and Cl^−^ ions) were added to mimic a 0.1 M concentration and electroneutrality (Table 1).

**TABLE 1:** Systems simulated in this study.

Protein	Membrane patch	Number of water molecules	Number of ions	Lipid species in membrane patch	Number of lipid molecules
PrP^C^ 2L39	PG patch	24127	492 NA 232 CL	POPG	258
				POPC	344
				Cholesterol	258
	SM patch	24389	232 NA 230 CL	POSM	258
				POPC	344
				Cholesterol	258
	PC patch	24127	232 NA	POPC	602
			230 CL	Cholesterol	258
Doppel 1I17	PG patch	25236	491 NA 233 CL	POPG	258
				POPC	344
				Cholesterol	258
	SM patch	25091	230 NA 230 CL	POSM	256
				POPC	343
				Cholesterol	257
	PC patch	25236	230 NA 230 CL	POPC	602
				Cholesterol	258

Each system was equilibrated following the protocol provided by CharmmGUI (energy minimization, step-wise position restraining, and equilibration). After equilibration, each trajectory was given a different set of initial velocities. Each trajectory is 2 microseconds long, with a timestep of 20 fs. The NPT ensemble was used for production simulations. Frames were saved at 500 ps intervals. A v-rescale thermostat (310 K) (Bussi et al., 2007) and semi-isotropic Parrinello-Rahman barostat (1 bar, 3 × 10^−4^/bar compressibility and 12 ps time constant) (Parrinello and Rahman, 1981) were used for production simulations. A cutoff of 1.1 nm was used for Lennard-Jones and Coulombic interactions. A reaction-field potential was applied to Coulomb interactions (Tironi et al., 1995). The dielectric constant was set to 2.5, as required by the water model.

The first 10% of each trajectory was discarded for analysis. Data analysis was done using GROMACS tools (Berendsen et al., 1995; Abraham et al., 2015), and Python scripts using the MDAnalysis (Michaud-Agrawal et al., 2011; Gowers et al., 2016) and LiPyphilic (Smith and Lorenz, 2021) libraries.

## Results

**FIGURE 2:**
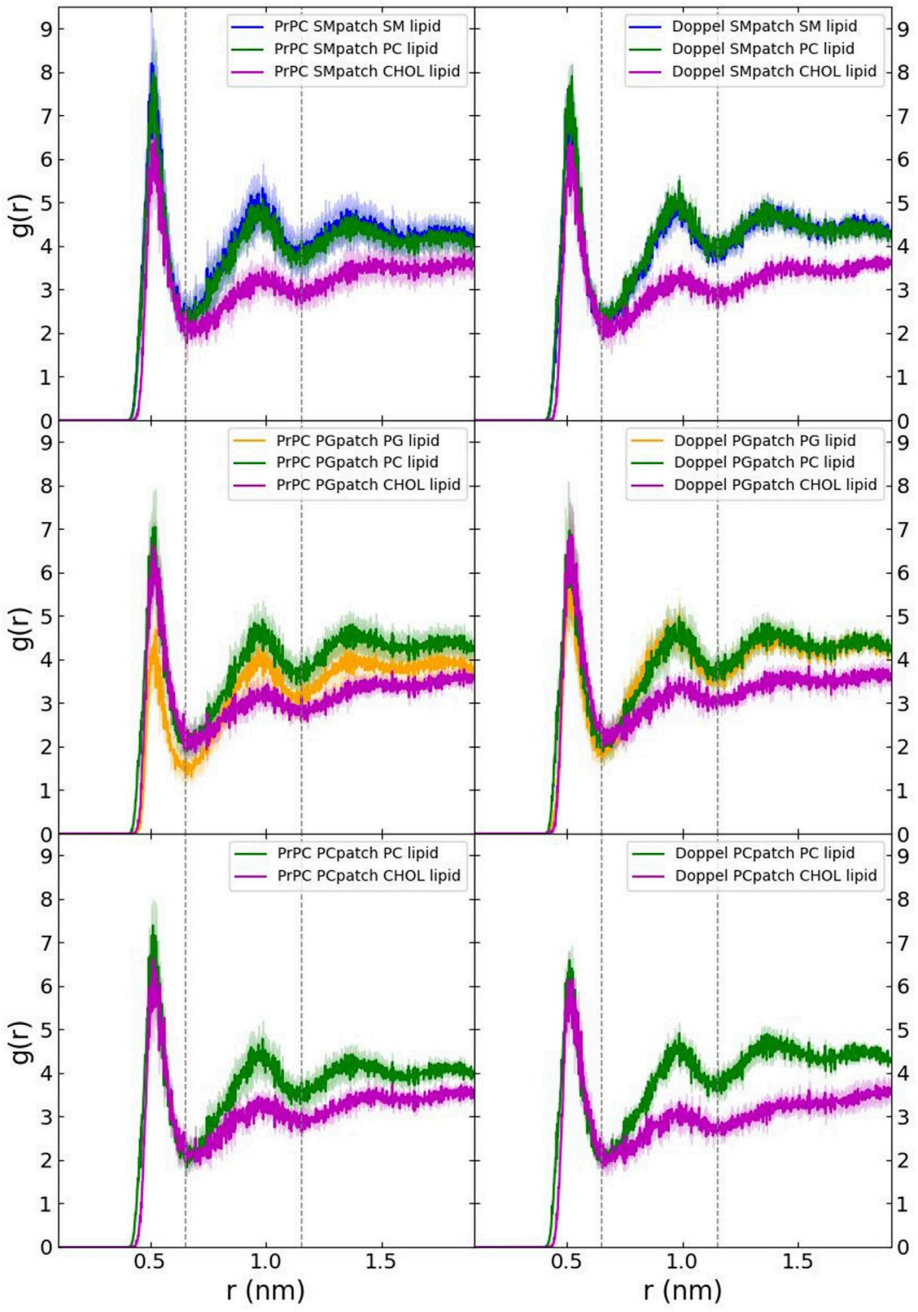
Radial distribution function of each lipid species with respect to the GPI anchor.

**FIGURE 3:**
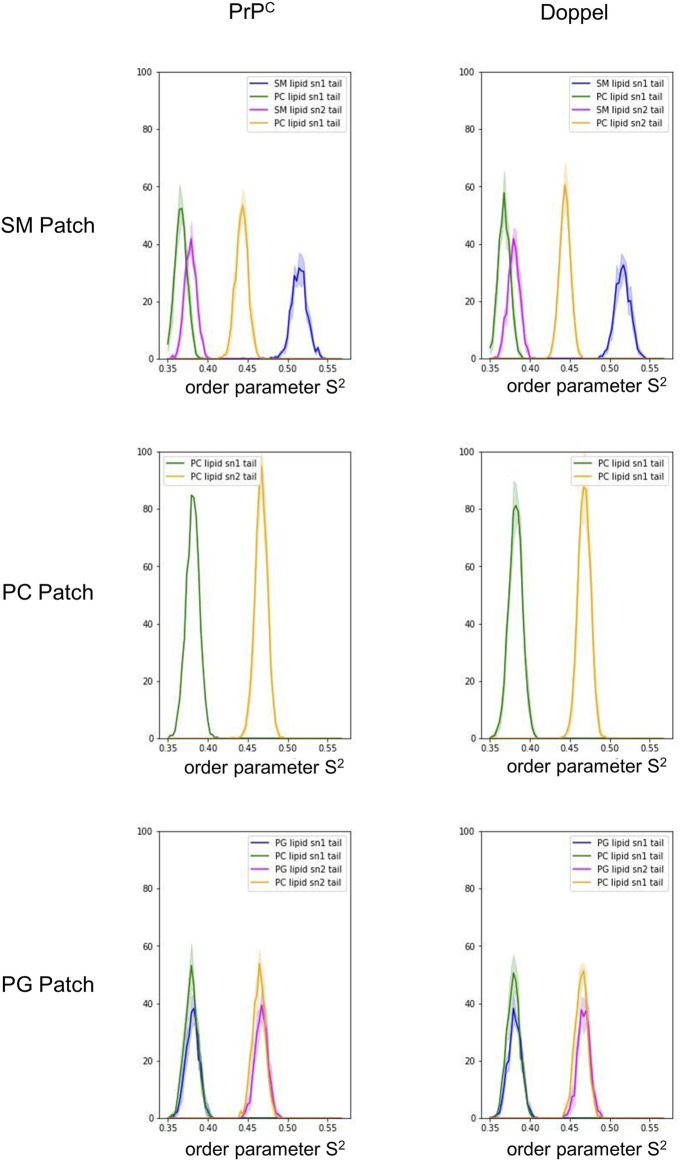
Histogram of the order parameter S^2^. The solid lines represent an average over the five trajectories in each membrane patch. The shade represents one standard deviation.

First, we examined the quality of the simulated membrane environments. The area per lipid and thickness of the membrane patches are consistent with the expected values of membranes simulated with the Martini forcefield (Table 2). We calculated the radial distribution function for each lipid species with respect to the GPI anchor molecule (Figure 2). The plots reveal consistent maxima and minima positions across all patches, suggesting equivalent lipid shells surrounding the GPI anchor in each set of simulations. Although we observed a slightly lower radial distribution function value in simulations of the Prion protein in the POPG patch, the position of the maxima and minima remained consistent with the other scenarios. To inspect the behavior of the lipid tails, we histogrammed the average order parameter S^2^ for each lipid tail (acyl chains for POPC and POPG, and sphingosine chain for POSM, Figure 3). The profiles are consistent across sets of simulations, indicating that the sampling quality of the lipids in the membrane patches is uniform for all trajectories.

**TABLE 2:** Average area per lipid and membrane thickness of the membrane patches.

Protein	Membrane patch	Area per lipid		Membrane thickness	
		average [Å^2^]	standard deviation [Å^2^]	average [Å^2^]	standard deviation [Å^2^]
PrP^C^	SM patch	51.34	0.02	30.81	0.01
	PG patch	51.68	0.02	31.70	0.01
	PC patch	51.54	0.03	31.73	0.01
Doppel	SM patch	51.32	0.01	30.83	0.01
	PG patch	51.67	0.01	31.70	0.01
	PC patch	51.52	0.01	31.75	0.01

The reported average for each system was obtained by averaging the averages from the five trajectories of each system.

### The globular domain of PrP^C^ exhibits well-defined modes of interaction with the membrane surface

**FIGURE 4:**
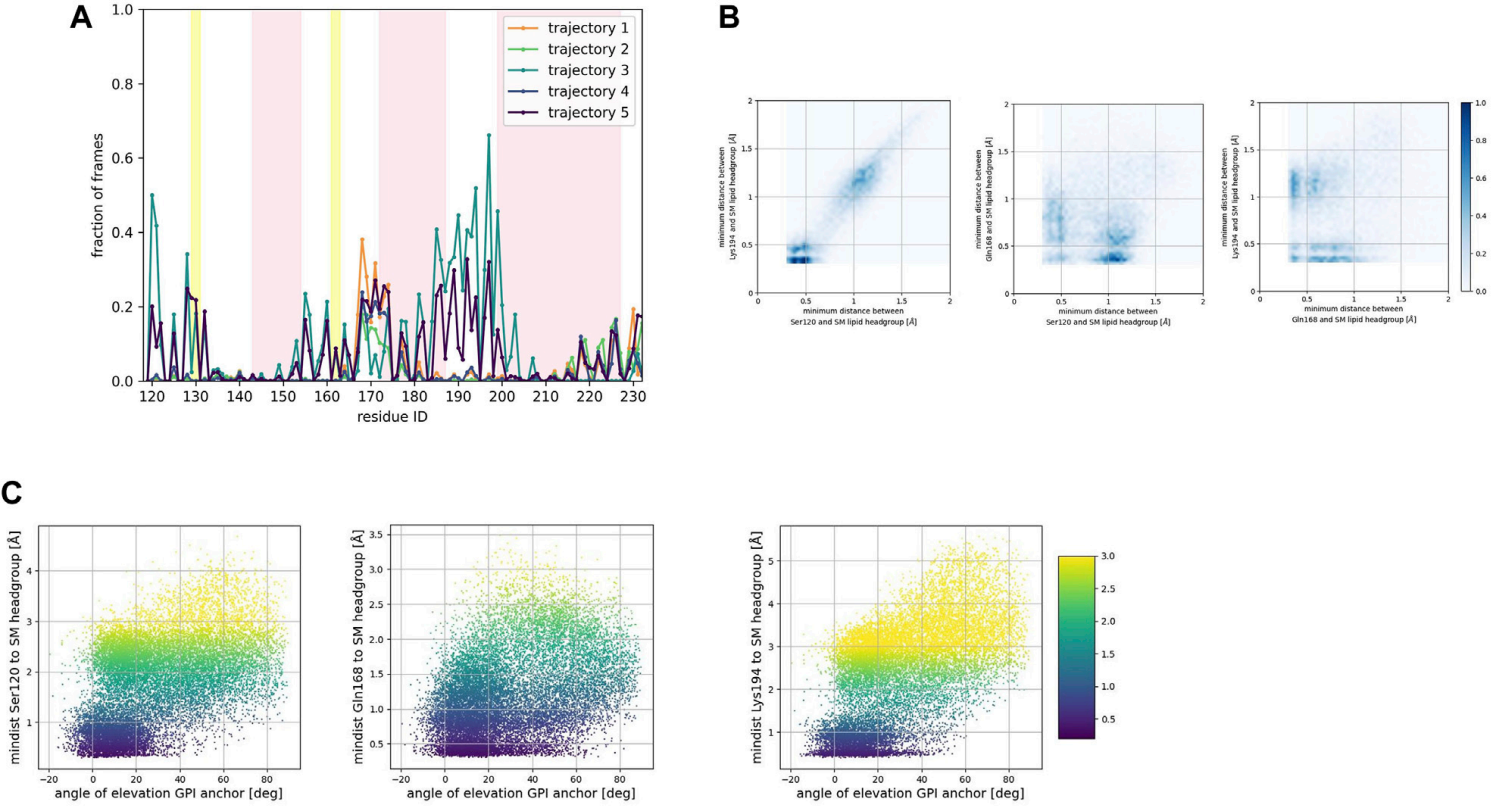
**(A)** Corresponds to the fraction of frames that show binding between the residue side chain and the SM headgroup. Binding was counted if the minimum distance between the side chain and the NC3 or PO4 beads of the SM lipid was less than 0.7 nm. **(B)** Shows 2D histograms of the minimum distance of residues representative of each binding site to the SM headgroup. The color bar represents the minimum distance between side chains and lipid headgroup, displayed in shades of blue; the darkest blue indicate distances equal to or greater than 1.0 nm. **(C)** Shows scatter plots of the minimum distance of residues representative of each binding site to the SM headgroup versus the angle of elevation of the GPI headgroup. The colorbar corresponds to the minimum distance values (any minimum distance greater than 3.0 nm was assigned yellow color).

**FIGURE 5:**
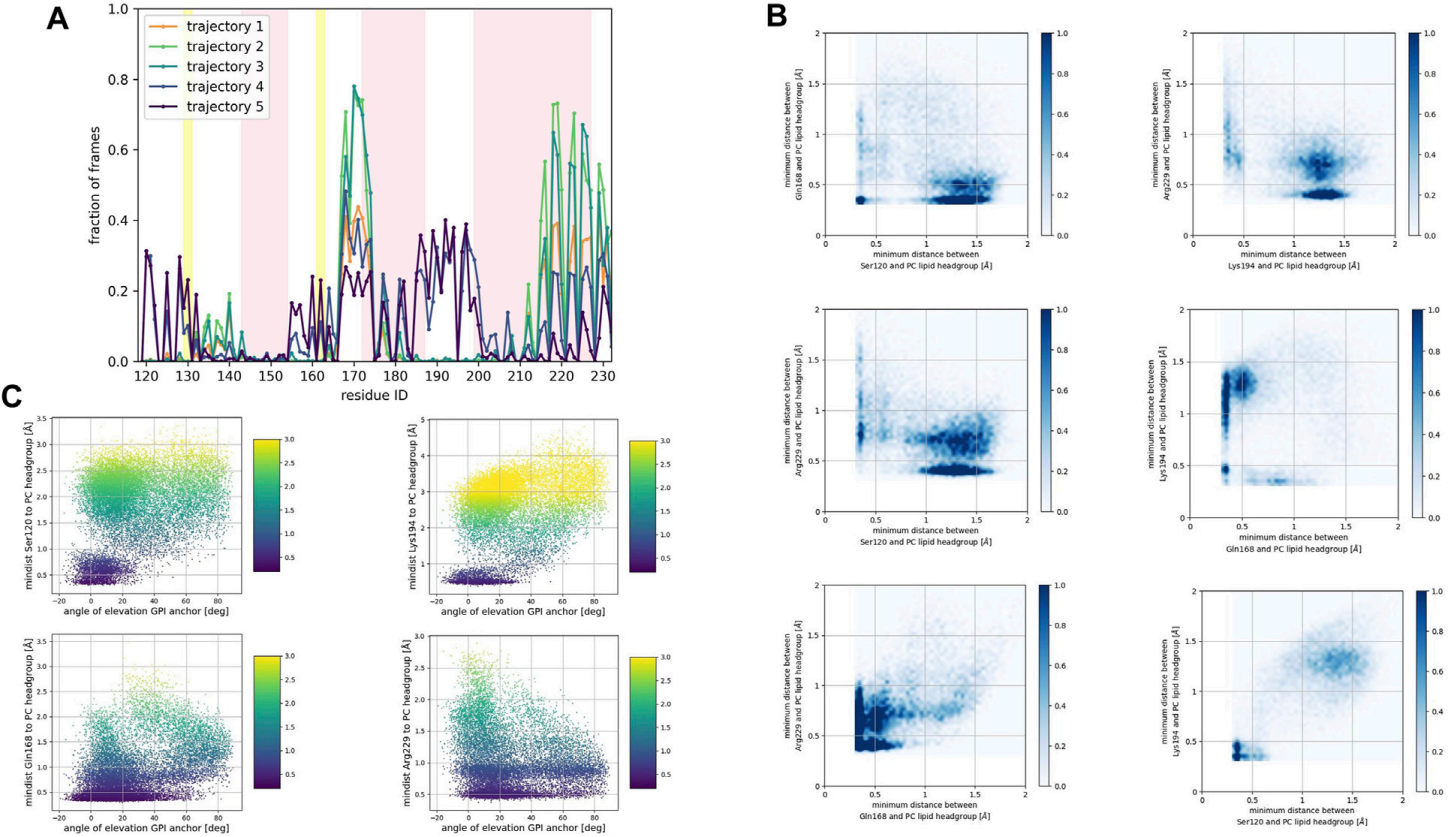
**(A)** Corresponds to the fraction of frames that show binding between the residue side chain and the PC headgroup. Binding was counted if the minimum distance between the side chain and the NC3 or PO4 beads of the PC lipid was less than 0.7 nm. **(B)** Shows 2D histograms of the minimum distance of residues representative of each binding site to the PC headgroup. The color bar represents the minimum distance between side chains and lipid headgroup, displayed in shades of blue; the darkest blue indicate distances equal to or greater than 1.0 nm. **(C)** Shows scatter plots of the minimum distance of residues representative of each binding site to the PC headgroup versus the angle of elevation of the GPI headgroup. The colorbar corresponds to the minimum distance values (any minimum distance greater than 3.0 nm was assigned yellow color).

**FIGURE 6:**
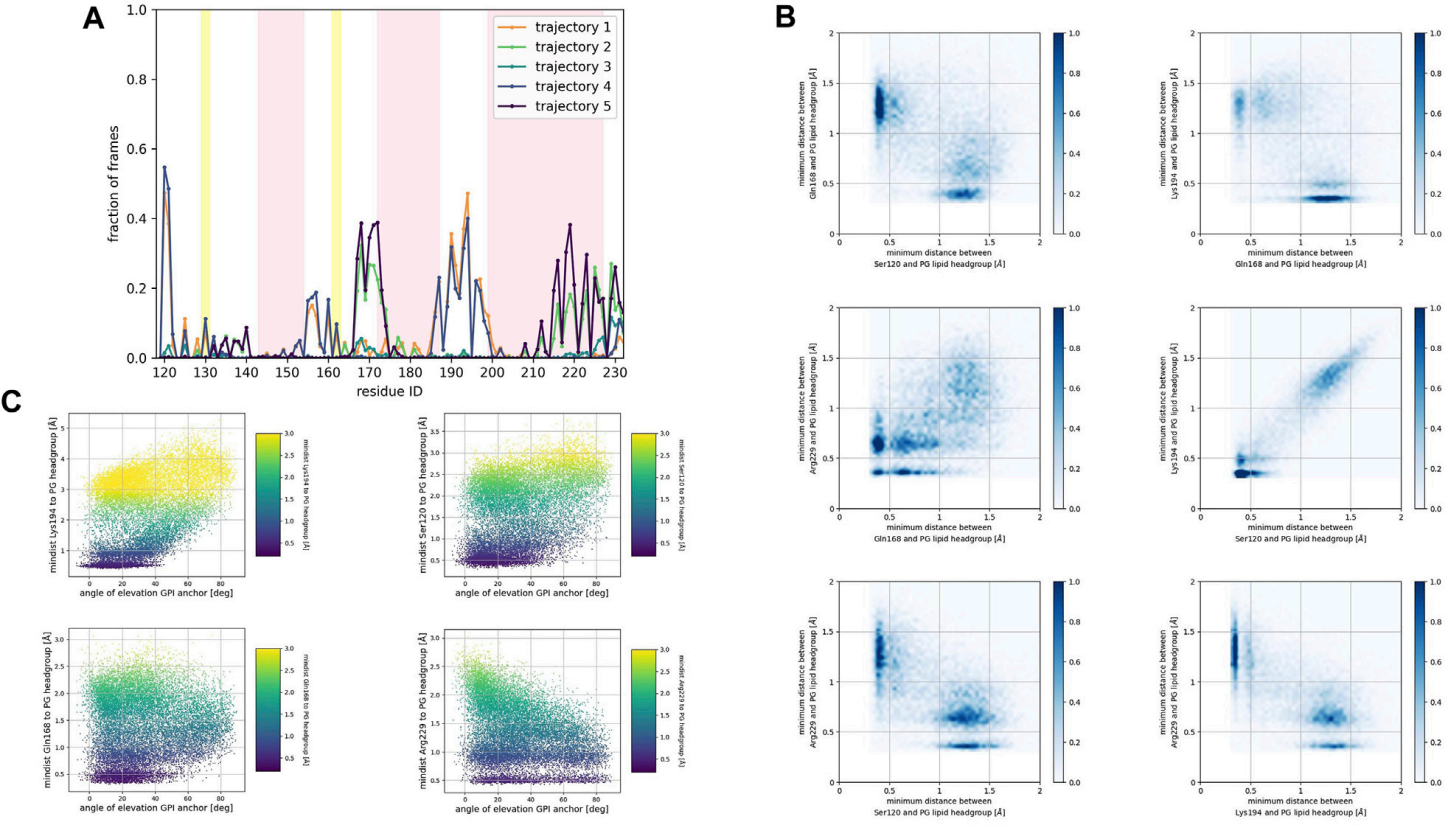
**(A)** Corresponds to the fraction of frames that show binding between the residue side chain and the PG headgroup. Binding was counted if the minimum distance between the side chain and the GL0 or PO4 beads of the PG lipid was less than 0.7 nm. **(B)** Shows 2D histograms of the minimum distance of residues representative of each binding site to the PG headgroup. The color bar represents the minimum distance between side chains and lipid headgroup, displayed in shades of blue; the darkest blue indicate distances equal to or greater than 1.0 nm. **(C)** Shows scatter plots of the minimum distance of residues representative of each binding site to the PG headgroup versus the angle of elevation of the GPI headgroup. The colorbar corresponds to the minimum distance values (any minimum distance greater than 3.0 nm was assigned yellow color).

To identify preferred protein residue-membrane interactions, we calculated the fraction of frames where a residue side chain was within 0.7 nm of the phospholipid head group. A cutoff distance of 0.7 nm is a standard descriptor of bound states between the side chain of peripheral proteins and membrane surfaces modeled with the Martini forcefield (Srinivasan et al., 2021). An interaction indicated binding if this fraction exceeded 0.3 in each trajectory for the SM patch (Figure 4A), the PC patch (Figure 5A), and the PG patch (Figure 6A). A cutoff fraction of 0.3 was chosen based on the analysis of the contacts distribution across all systems. Our analysis revealed no association between α-helix 1 and the membrane surface in all cases of the globular PrP^C^. Similarly, no association was observed between the N-terminus of α−helix 3 and the cell membrane.

Consistently across all three patches, we identified the β2−α2 loop (Gln168, Ser170, Asn171, Gln172, Asn173) and the α2−α3 loop (Thr191, Thr192, Thr193, Lys194, Asn197) as dominant fragments of globular PrP^C^ that favorably interact with the surface of the membrane patches. A third fragment corresponding to the N-terminus of the globular domain of PrP^C^, also interacts favorably. However, only a small number of frames show such a binding event. In the PC patch and PG patch simulations only, we observed association events between the C-terminus of α−helix 3 and the membrane surface.

To characterize the binding sites, we first examined whether they occur simultaneously (Figures 4B, 5B, 6B). 2D histograms reveal that the binding site corresponding to the N- terminus of the globular domain of the Prion protein and the α2−α3 loop exhibit simultaneous occurrence. This observation makes sense in light of our previous findings (Soto et al., 2023) that demonstrate the dynamic coupling of these two fragments.

The β2−α2 loop did not exhibit concurrent binding with any other fragment in the SM and the PG patches (Figures 4B, 6B). In the PC patch simulations, however, we observed a minimal population of β2−α2 loop interacting with the membrane surface concurrently as the N-terminus of the globular domain and the α2−α3 loop do (Figure 5B).

In both the PC patch and PG patch simulations, we observed the C-terminus of α−helix 3 associating with the membrane surface (Figures 5B, 6B). Although less pronounced than the correlation between the N-terminus of the globular domain and the α2−α3 loop, a notable overlap of binding mode occurrence with the β2−α2 loop was evident.

To assess the influence of the GPI anchor on the protein-membrane association, we constructed scatter plots correlating the distance of each residue side chain to the phospholipid head group with the angle of elevation of the GPI anchor. This angle was measured as the angle of elevation of a vector that spans the headgroup of the GPI anchor molecule (Figures 4C, 5C, 6C).

The plots show no significant correlation between the angle of elevation and the binding of the β2−α2 loop or the C-terminus of α−helix 3 to the membrane surface. This observation aligns with the previously identified binding correlation between the β2−α2 loop and the C-terminus of α−helix 3, suggesting that the orientation of the GPI anchor head group does not strongly dictate their association with the membrane surface.

In contrast, a pattern emerged indicating a correlation between the angle of elevation of the GPI anchor head group and the binding of the N-terminus of globular PrP^C^. A similar, though more spread, correlation was observed between the GPI anchor angle of elevation and the binding of the α2−α3 loop. These findings support the interpretation that the two sites may correspond to a single binding mode influenced by the steric constraints imposed by GPI anchoring.

We monitored the same number of trajectories for the Doppel protein as the Prion protein, using identical membrane environments and GPI molecule. Similar to the Prion protein, our analysis revealed no association between α−helix 1 of Doppel and the membrane surface. In contrast to PrP^C^, we observed a sparsely populated binding mode (fraction of frames of at most 0.1) corresponding to the β2−α2 loop in Doppel in the simulations performed in the SM and the PC patches (Supplementary Figures S1A, B). We speculate that sampling these binding modes for the Doppel protein might not be enough in our simulations.

In the PG patch simulations, the binding modes of the Doppel protein resemble to some extent the modes found in the PrP^C^ simulations (Supplementary Figure S1C). However, the descriptor we used to identify binding (fraction of frames at which the minimum distance from the side chain to the lipid headgroups is less than 0.7 nm) displays lower values in the Doppel protein. One binding mode involves the β2−α2 loop (driven by residue Ser96 and Asn99). We observed a larger fraction of frames corresponding to a binding mode involving the second half of α−helix 2 (Asn111, Gln114), the short α−helix 2′ (Gln118, Ser122, Lys125, Gln126), and the N-terminus of α−helix 3 (Lys129). The largest number of residues in the binding mode are located in α−helix 2′, fragment that would correspond to the α2−α3 loop in PrP^C^. The type of amino acids in the binding mode is similar in both proteins: polar amidic, polar hydroxylic, and basic. However, threonine amino acids are not involved in the binding due to being buried in the inside of the Doppel protein conformation.

### Membrane response to protein association

**FIGURE 7:**
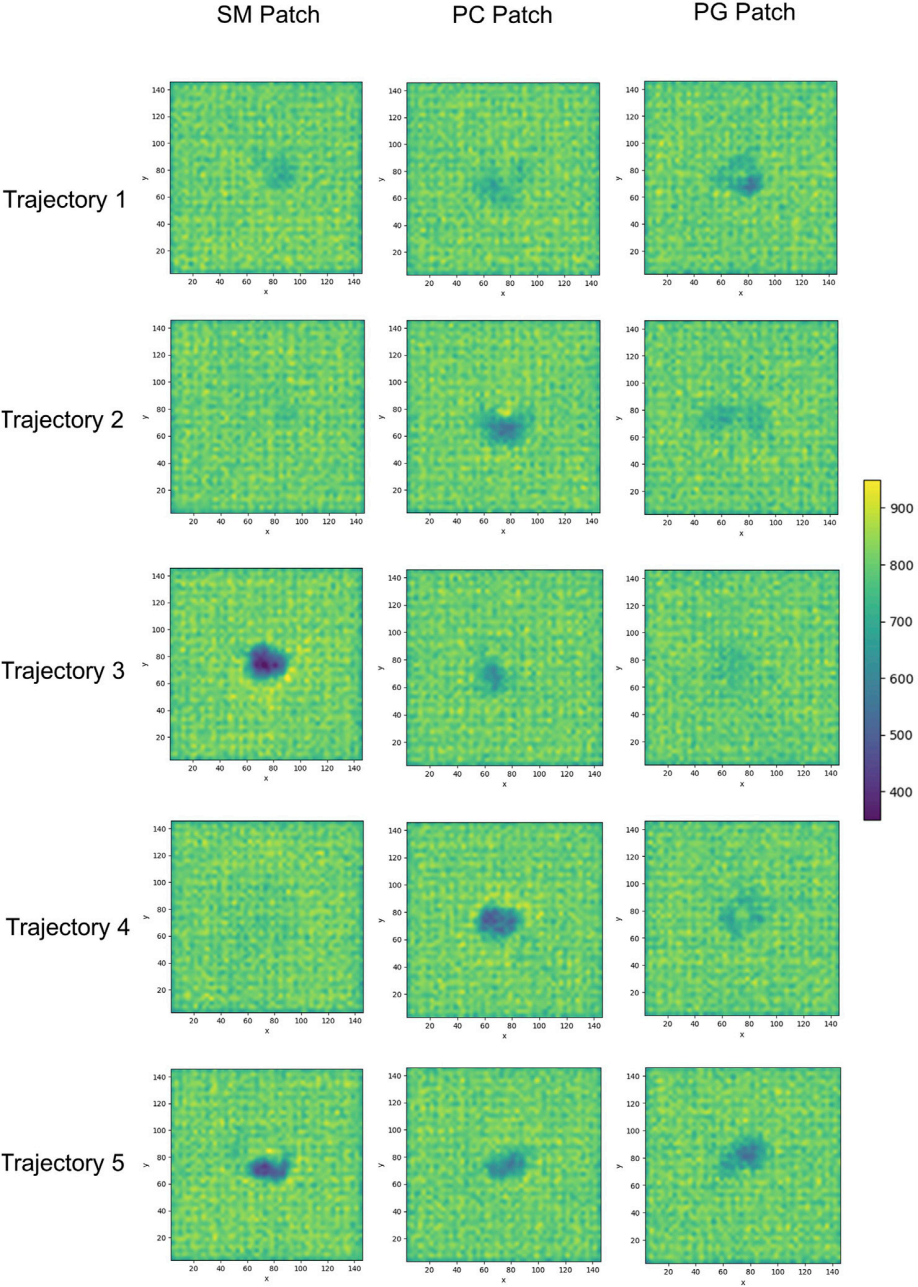
2D cumulative histograms of the beads representing the headgroup of SM and PC lipids in the SM patch, PC lipid in the PC patch, and PG and PC lipids in the PG patch.

We examined the membrane surface response to protein association by plotting the density of lipid head groups for each membrane patch. In some instances, the plots revealed the formation of depressions or clefts on the membrane surface, which we call “divots” only in the globular PrP^C^ trajectories. While the size of these divots varied, they typically displayed an oval shape, with an average larger axis of approximately 10 Å (Figure 7).

We calculated the number density for residues identified as part of the binding sites to assess the insertion depth of protein side chains into these divots (Figure 9). In the figure, the dashed line represents the number density profile of the cholesterol head group, which indicates side chain penetration depth, as cholesterol head groups are located below the membrane surface.

**FIGURE 8:**
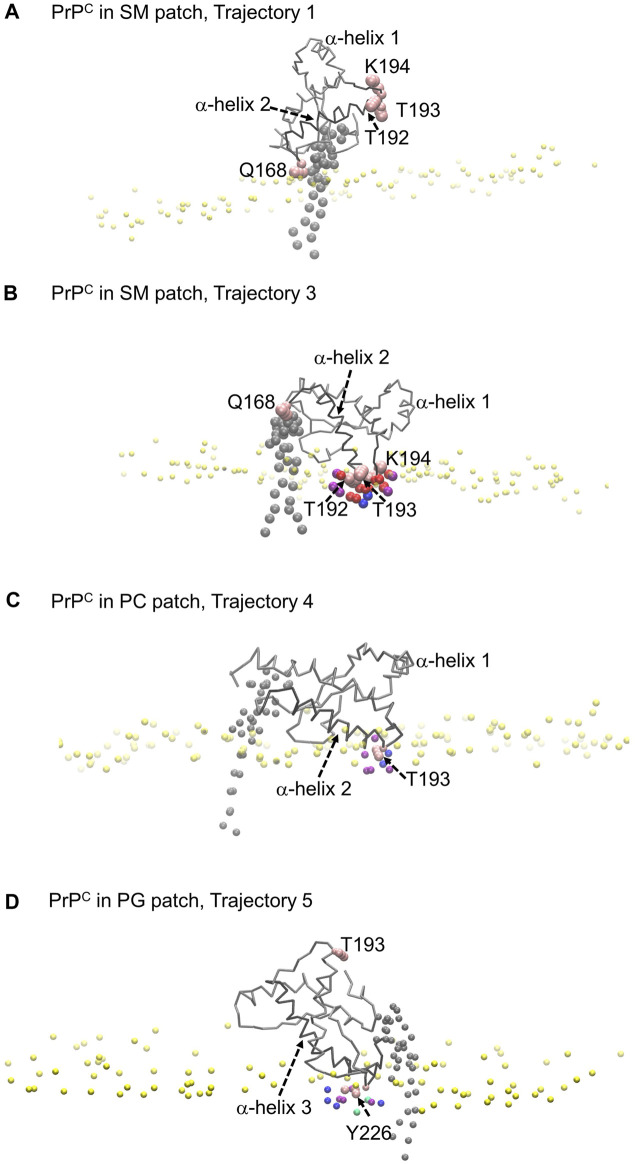
Modes of PrP^C^ interaction with membrane patch. Purple beads represent POPC beads close to inserted side chains; blue beads represent cholesterol beads close to inserted side chains; red beads represent POSM beads close to the inserted side chains; green beads represent POPG beads close to the inserted side chains. **(A)** PrP^C^ in SM patch shows Gln168 interacting with the membrane patch surface, but side chain insertion is not measurable. The tilting of α-helix 2 sets the α2–α3 loop away from the membrane surface. **(B)** PrP^C^ in SM patch shows T192 and T193 inserted in the surface of the membrane. The tilting of α-helix 2 sets the β2–α2 loop away from the surface. **(C)** PrP^C^ in PC patch shows T193 inserted in the surface of the membrane. **(D)** PrP^C^ in PG patch shows Tyr226 inserted in the surface of the membrane. The tilting of α-helix 3 sets the α2–α3 loop away from the membrane surface.

The profiles show that Glutamine 168 and Asparagine 197 do not exhibit insertion into the membrane (Figure 8A). Instead, these two residues interact mainly with the head groups of phospholipids and sphingomyelin but do not significantly penetrate the surface. In other words, in cases where we observe the association of these two residues with phospholipid head groups, measurable divots were not observed.

**FIGURE 9:**
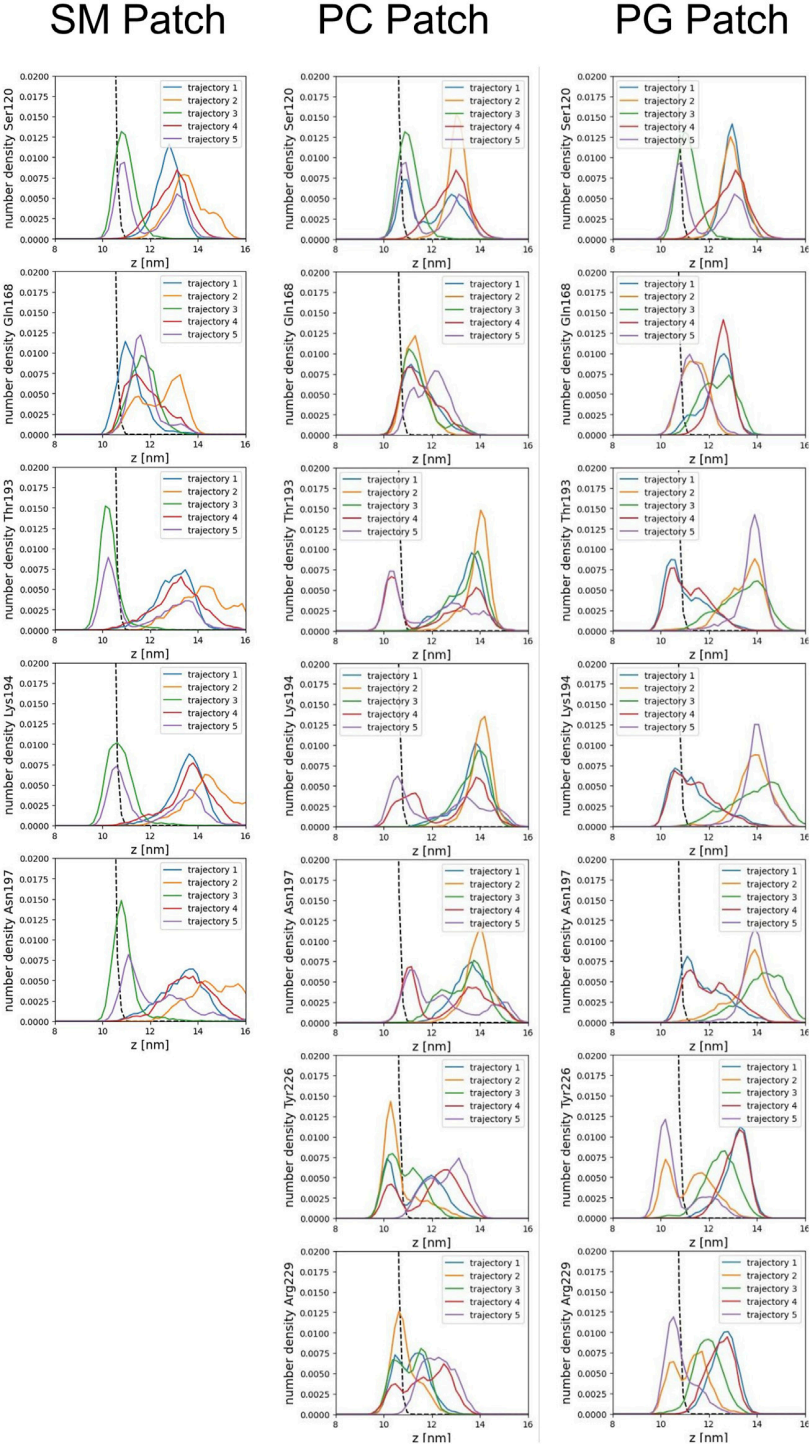
Number density profiles of residue side chains representative of binding sites. The dashed line corresponds to the number density profile of the headgroup bead of cholesterol.

Our analysis across all patches reveals a strong correlation between the presence of divots and the insertion of threonine residues in the α2−α3 loop of the Prion protein (Figures 8B, C). The number density profiles of threonine 193 consistently showed the highest degree of insertion across all patches (Figure 9). Only in the PC patch and PG patch, we observe measurable insertion of Lysine 194, with a much smaller fraction of frames exhibiting insertion in the SM patch. However, compared to threonine, Lysine 194 showed shallower insertion (Figure 8B).

In the PC and PG patches, we also observed the insertion of residues from the C-terminus of α−helix 3 of globular PrP^C^. Although more binding events were observed in the PC patch, both patches displayed the insertion of Tyrosine 226 and Arginine 229 in about the same proportion (Figure 8D). Interestingly, the binding of the C terminus of α−helix 3 correlates with the binding of the β2−α2 loop. However, the residues in the loop do not insert deeply into the membrane. In the PG patch, we identified only one trajectory showing deep insertion of Serine 120, which was not observed to the same extent in other patches. Although protein association of the N-terminus of the globular domain and the α2−α3 loop are correlated, threonine residues in the α2−α3 loop are the primary contributors to insertion in the membrane surface. We did not observe insertion of the GPI headgroup on the membrane surface (Supplementary Figure S2).

The residue side chain insertions tend to occur at relatively shallow depths. We note that inserted side chains interact with beads representing SM, PC, and PG head groups, phosphate groups, glycerol groups, and AM beads of sphingomyelin, and the hydroxyl (OH) group of cholesterol, within the divots. We conclude that electrostatic interactions drive the protein-lipid interactions. The infrequent interactions with beads representing atoms in the lipid acyl chains we observed come from a steric hindrance response.

**FIGURE 10:**
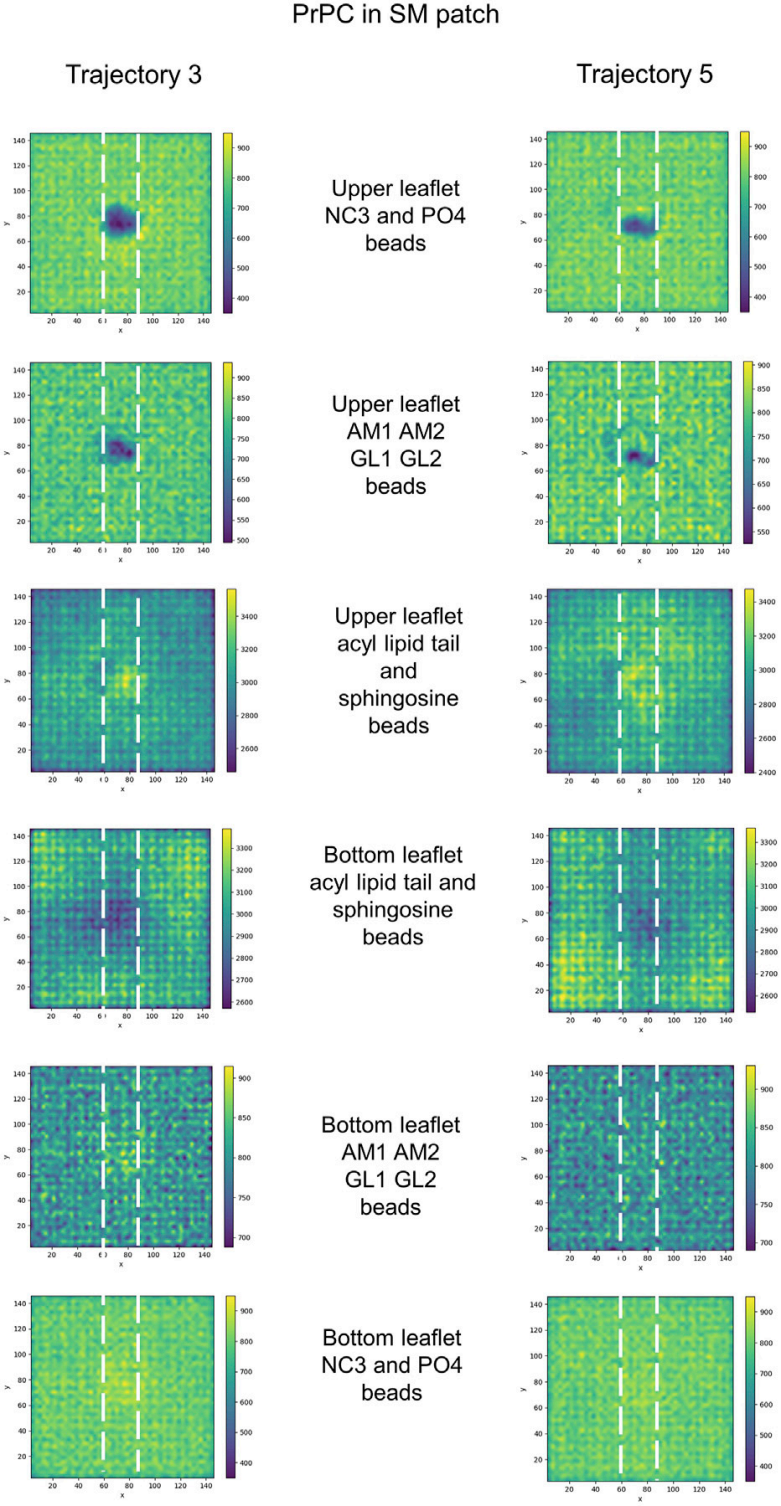
2D cumulative histograms only for SM patch trajectories showing well-defined divots. Each row corresponds to the 2D cumulative histogram of a set of beads in the upper and bottom leaflets as labeled in the figure.

Further examination of the membrane patches in the divot region revealed that the beads corresponding to head groups and phosphate groups tilted away (from the vertical) to accommodate side chain insertion. Similar tilting was observed for beads representing glycol groups in the phospholipids and for AM beads in sphingomyelin. In the SM patch and as a result of the tilting of the headgroups, we detected evidence of lipid tails tilting inward under the divot (Figure 10). However, the other patches did not show this effect (Supplementary Figures S3–S6). Notably, in trajectories where divots formed in the SM patch, we observed coupling between the lipid tails of the upper leaflet and those of the lower leaflet. However, no measurable divot formation occurred on the surface of the lower leaflet. We did not observe measurable divots on the surface of the PG patch upon Doppel protein binding (see Supplementary Figure S7). The binding site, rich in amidic, two lysine, and one serine residues, inserts shallowly on the surface.

## Discussion

The first building block of a structural model of Prion protein conformational conversion and propagation necessitates a picture of mature PrP^C^ interacting with the surface of the plasma membrane. Our simulations show that globular PrP^C^ associates with the membrane surface more specifically than Doppel protein due to coupled enthalpic and entropic effects. The specific protein sequence that binds to the surface of the membrane elicits a distinct membrane response. The binding of globular PrP^C^ is observed in the three membrane patches we studied, but Doppel binding is observed only in the patch enriched with the anionic phospholipid POPG. In response to globular PrP^C^ binding, the membrane surface re-arranges locally into small divots that accommodate the inserted side chains. No similar response could be measured in the Doppel protein binding events. Polar residue side chains containing the -NH_2_ group (Gln and Asn) do not insert deeply into the membrane.

In contrast, polar residue side chains containing the -OH group (Thr, Ser, and Tyr) insert to a greater extent on the membrane even in the case of Tyr where an aromatic ring imposes steric hindrance. Positively charged side chains (Lys and Arg) insert on the membrane, although to a lesser extent than polar side chains with the -OH group. The charged side chains may interact more favorably with the negatively charged phosphate groups. Located in the head groups, thus presenting shallower insertion.

The protein sequence, topology, and GPI-anchoring govern the association modes of globular PrP^C^ and Doppel proteins to the membrane surface. Our simulations showed no binding event involving α−helix 1 for any protein. The residue sequence of a-helix 1 in PrP^C^ has four charged side chains that Doppel does not have, however sequence alignment shows similarities (**D**WED**RYY**RE**NMY**R in PrP^C^ and **E**-GN**RYY**AA**NYW**Q in Doppel). Although the electrostatics pattern of α−helix 1 in PrP^C^ is richer, we speculate that steric constraints derived from protein topology and anchoring of the globular proteins prevents α−helix 1 from reaching the surface of the membrane. A similar reasoning applies for the lack of association between each the N-terminus of α−helix 3 in PrP^C^ and α−helix 3 in Doppel, and the membrane surface (ETDV**KMM**E**RVV**EQ**MC** in PrP^C^ and KLHQ**RVL**W**RLI**KE**IC** in Doppel).

In globular PrP^C^, GPI-anchoring favors the association of the α2−α3 loop (T**V**TT**T**TKGENFT) with the membrane surface in the three patches we studied. Our simulations sampled only binding of α−helix 2' (NQAEFSREKQ) to the patch rich in anionic phospholipids in the Doppel protein. Although the response elicited on the membrane surface is distinct in each case, it is interesting to note that in the topology of globular PrP^C^, the α2−α3 loop corresponds to α−helix 2′ in Doppel. Although protein-lipid interactions are different in each case, constraints derived from topology may contribute to binding.

In globular PrP^C^, the association between each the β2−α2 loop and the C-terminus of α−helix 3 and the membrane surface appears to be governed more by protein sequence rather than the GPI anchor. An equivalent binding mode was not observed in the Doppel simulations. We speculate that while both proteins are GPI-anchored to sphingomyelin and cholesterol-rich domains, interactions of with the membrane may be influenced by details of the molecular microenvironment. Also, differences in GPI anchor sequence may modulate such interactions.

Our coarse grain modeling, although informative about overall features of protein-membrane association, cannot provide a quantification of the lifetime of the binding modes or membrane surface divots we observed. The extent of the sampling of our simulations (2 microseconds long trajectories, 5 trajectories per patch, for a total of 15 trajectories per protein) might not have sampled enough all possible binding sites or membrane response. Therefore, we cannot quantify the relative populations of each binding mode. Our study did not address the effect of glycosylation on protein-membrane interactions, which has been well-documented already (DeMarco and Daggett, 2009; Wu et al., 2015).

Intriguingly, the two main binding modes we observed in globular PrP^C^ involve protein regions critical for misfolding and aggregation. The β2−α2 loop has been characterized as an amyloidogenic fragment (Thompson et al., 2006), as a site that regulates propensity to misfolding (Soto et al., 2021), and as a loop that modulates transmission barrier in prion diseases (Gorfe and Caflisch, 2007; Sigurdson et al., 2010). We speculate that when the β2−α2 loop interacts with the membrane surface, the fragment is protected from interacting with other nearby Prion proteins, PrP^C^ or PrP^Sc^, thus decreasing the likelihood of recognition and subsequent aggregation. The α2−α3 loop has been proposed as an initiation site of spontaneous protein misfolding based on fluorescence resonance energy transfer experiments (Sengupta and Udgaonkar, 2019). We argue that binding of the α2−α3 loop to the membrane protects the conformational integrity of the loop, preventing the amplification of conformational resonances (Soto et al., 2023) and thus avoiding misfolding. In an alternative scenario, when the loop is inserted in the membrane, an unexpected interaction might conformationally perturb the loop and initiate misfolding, affecting other protein regions connected by distal dynamic couplings.

Comprehensively characterizing the interaction modes between the Prion protein and the membrane surface under physiological conditions establishes a fundamental reference point for understanding Prion protein conformational conversion (Mercer and Harris, 2022). The binding sites we identified are also key protein loops for misfolding and aggregation, which contributes to the understanding that conformational conversion is rare. The influence of GPI-anchoring competes with protein-lipid interactions to dictate membrane binding. Although we observe specific protein residues interacting with the membrane, we do not have evidence indicating that membrane composition affects binding. Instead, distinct membrane compositions show relatively similar conformational responses to protein binding.

We expect our work will inspire experimental studies using atomic force microscopy and spectroscopy techniques to investigate further effects of membrane remodeling induced by the Prion protein. Future investigations can characterize the membrane environment surrounding misfolded Prion proteins and Prion protein fibrils in pathological conditions. Interestingly, recently cryoEM-resolved three-dimensional structures of fibrillar aggregates of PrP^Sc^ include the residues of the globular C-terminus of PrP^C^, and some also include the residues from the polybasic and consecutive hydrophobic fragment (Telling, 2022). In such structures, the residues that form the α2-α3 loop in PrP^C^ are located on the end side of a lobe. Studies may focus on the effect of such region on fibril-membrane interactions and investigate whether the stretch of threonine residues induces a response on the membrane surface similar to the divots we observed. This will provide a crucial foundation for unraveling the complexities of the mechanisms of membrane disruption by fibrillar PrP^Sc^ in the context of prion diseases.

## Data Availability

The raw data supporting the conclusion of this article will be made available by the authors, without undue reservation.
