# Supplementary material for: Protein-lipid interactions and protein anchoring modulate the modes of association of the globular domain of the Prion protein and Doppel protein to model membrane patches
Source: Front Bioinform. 2024 Jan 5;3:1321287. doi: 10.3389/fbinf.2023.1321287 (PMC10796588; doi:10.3389/fbinf.2023.1321287)
Supplement: Supplementary file 1 [file Table1.pdf]

**Protein**	**Membrane patch**	**Lipid species in membrane patch**	**Number of lipid molecules**	**Number of water molecules**	**Number of ions**
PrP^C	PG patch	POPG	258	24127	492 NA
2L39		POPC	344		232 CL
		Cholesterol	258		
	SM patch	POSM	258	24389	232 NA
		POPC	344		230 CL
		Cholesterol	258		
	PC patch	POPC	602	24127	232 NA
		Cholesterol	258		230 CL
Doppel	PG patch	POPG	258	25236	491 NA
1I17		POPC	344		233 CL
		Cholesterol	258		
	SM patch	POSM	256	25091	230 NA
		POPC	343		230 CL
		Cholesterol	257		
	PC patch	POPC	602	25236	230 NA
		Cholesterol	258		230 CL

Table 1

Systems simulated in this study.
