# Supplementary material for: Protein-lipid interactions and protein anchoring modulate the modes of association of the globular domain of the Prion protein and Doppel protein to model membrane patches
Source: Front Bioinform. 2024 Jan 5;3:1321287. doi: 10.3389/fbinf.2023.1321287 (PMC10796588; doi:10.3389/fbinf.2023.1321287)
Supplement: Supplementary file 2 [file DataSheet1.PDF]

Supplementary information

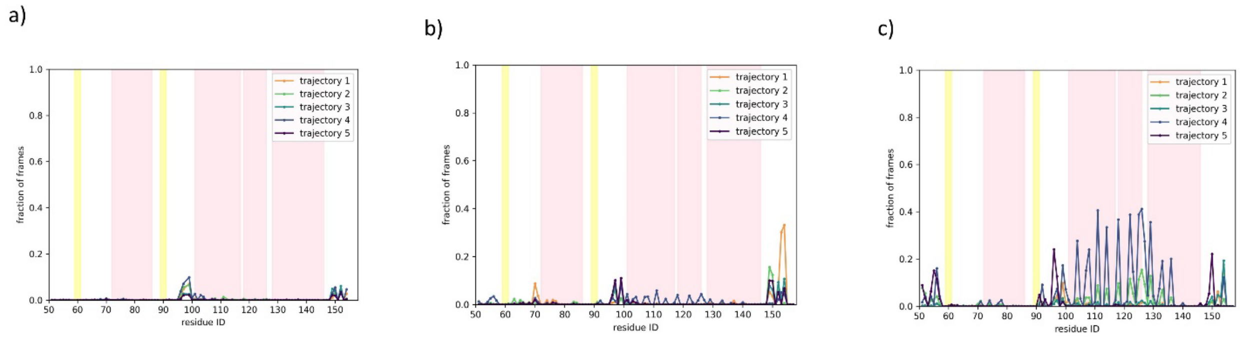

SI Figure 1.

Fraction of frames that show binding between the residue side chain and the SM headgroup (a), the PC headgroup (b), and the PG headgroup (c). Binding was counted if the minimum distance between the side chain and the headgroup beads of the lipid was less than 0.7 nm.

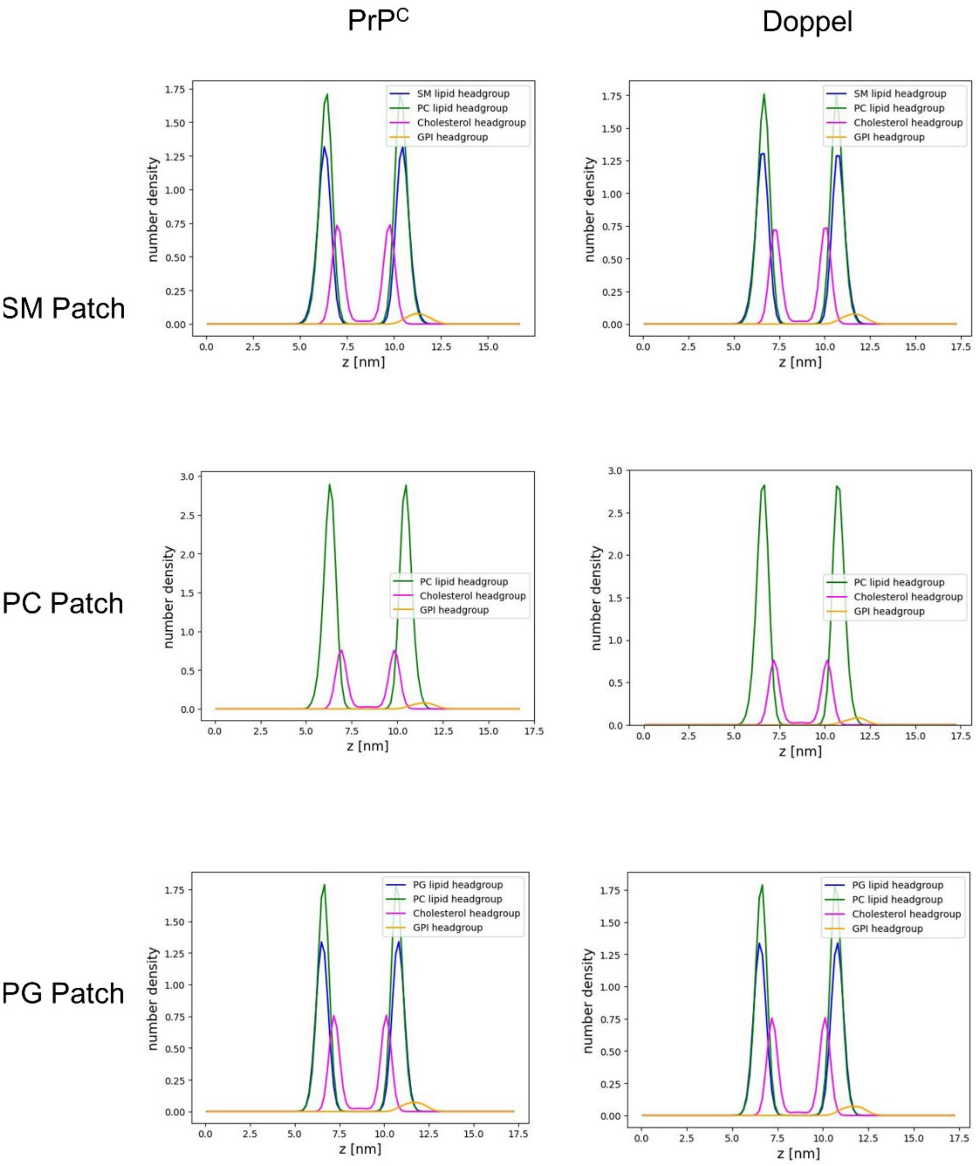

SI Figure 2.

Number density of each lipid and GPI molecule for each protein in each patch. The calculation was done for all concatenated trajectories for each patch.

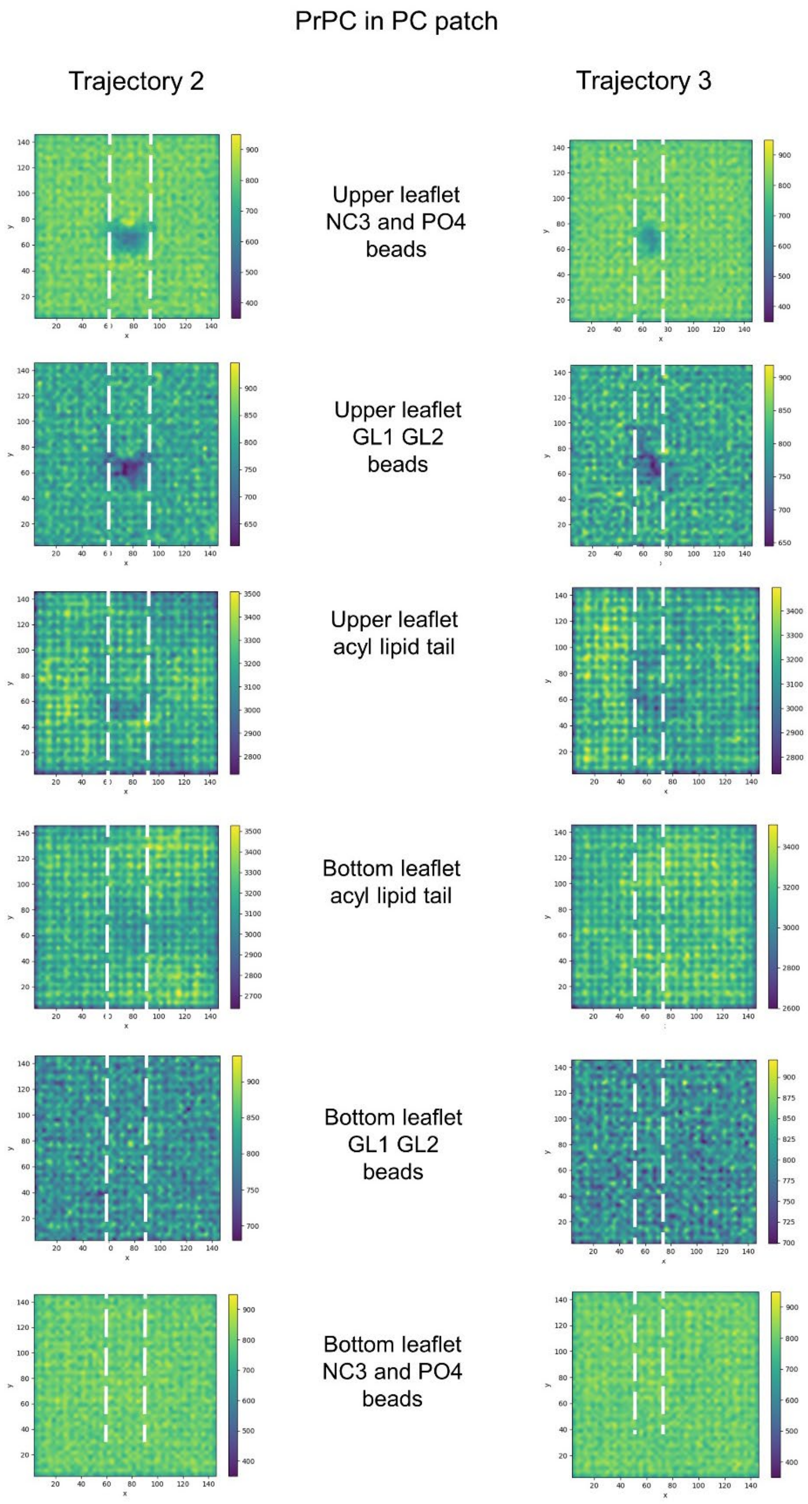

SI Figure 3

2D cumulative histograms only for globular PrP^{C} in PC patch trajectories 2 and 3 showing well-defined divots. Each row corresponds to the 2D cumulative histogram of a set of beads in the upper and bottom leaflets as labeled in the figure. Trajectory 1 did not show a well-defined divot.

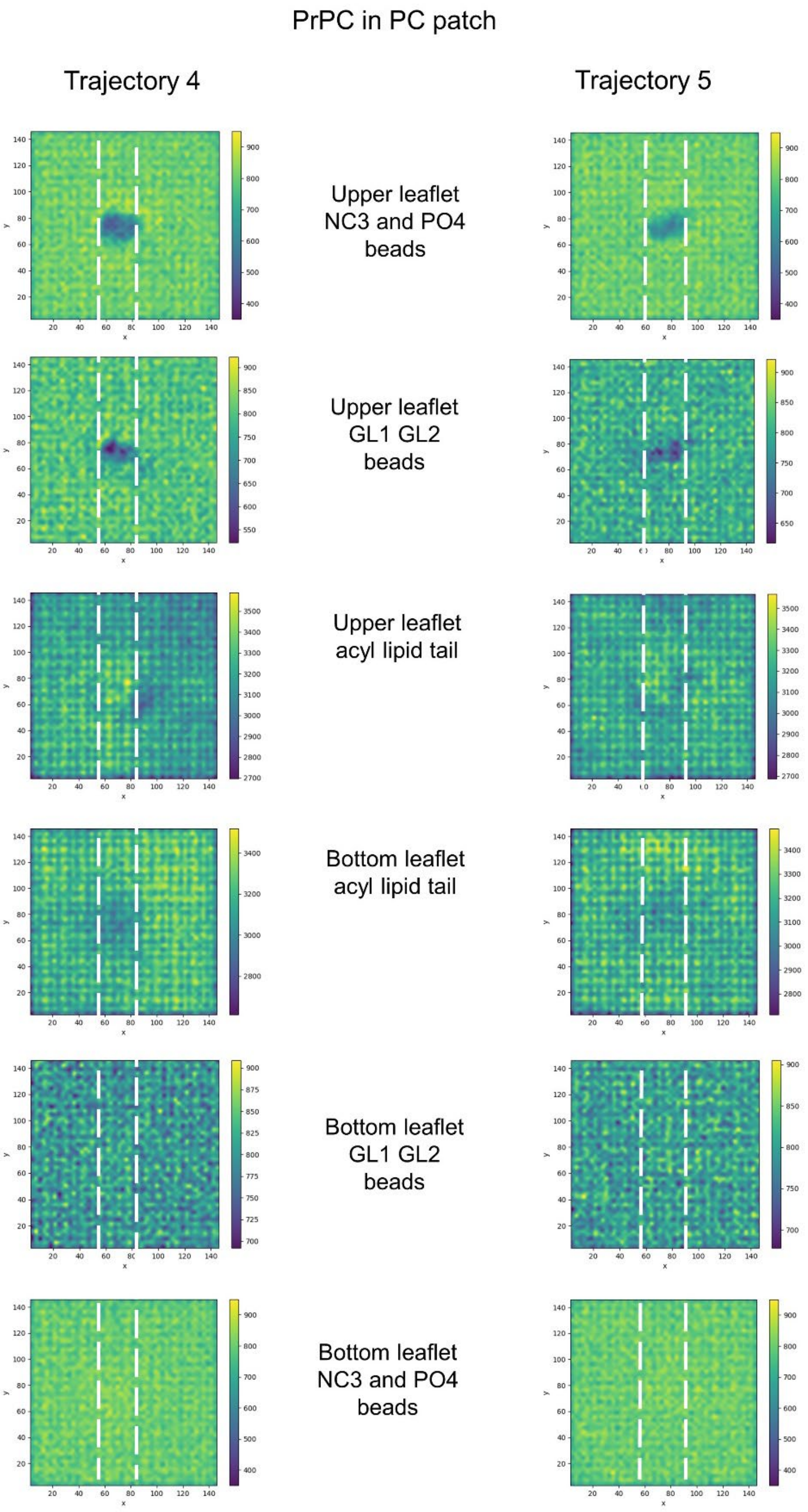

SI Figure 4

2D cumulative histograms only for globular PrP^C in PC patch trajectories 4 and 5 showing well-defined divots. Each row corresponds to the 2D cumulative histogram of a set of beads in the upper and bottom leaflets as labeled in the figure.

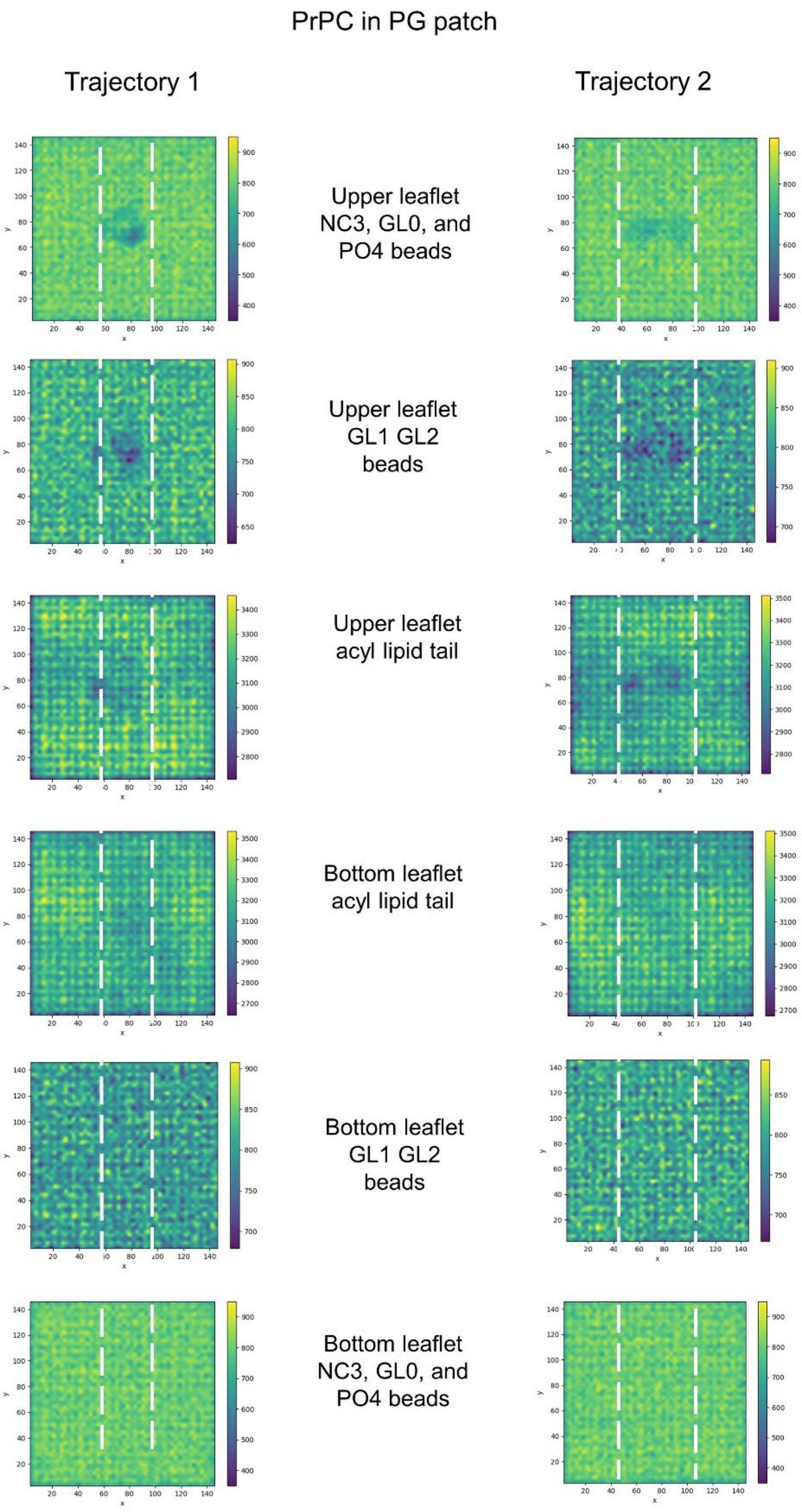

SI Figure 5

2D cumulative histograms only for globular PrP^{C} in PG patch trajectories 1 and 2 showing well-defined divots. Each row corresponds to the 2D cumulative histogram of a set of beads in the upper and bottom leaflets as labeled in the figure.

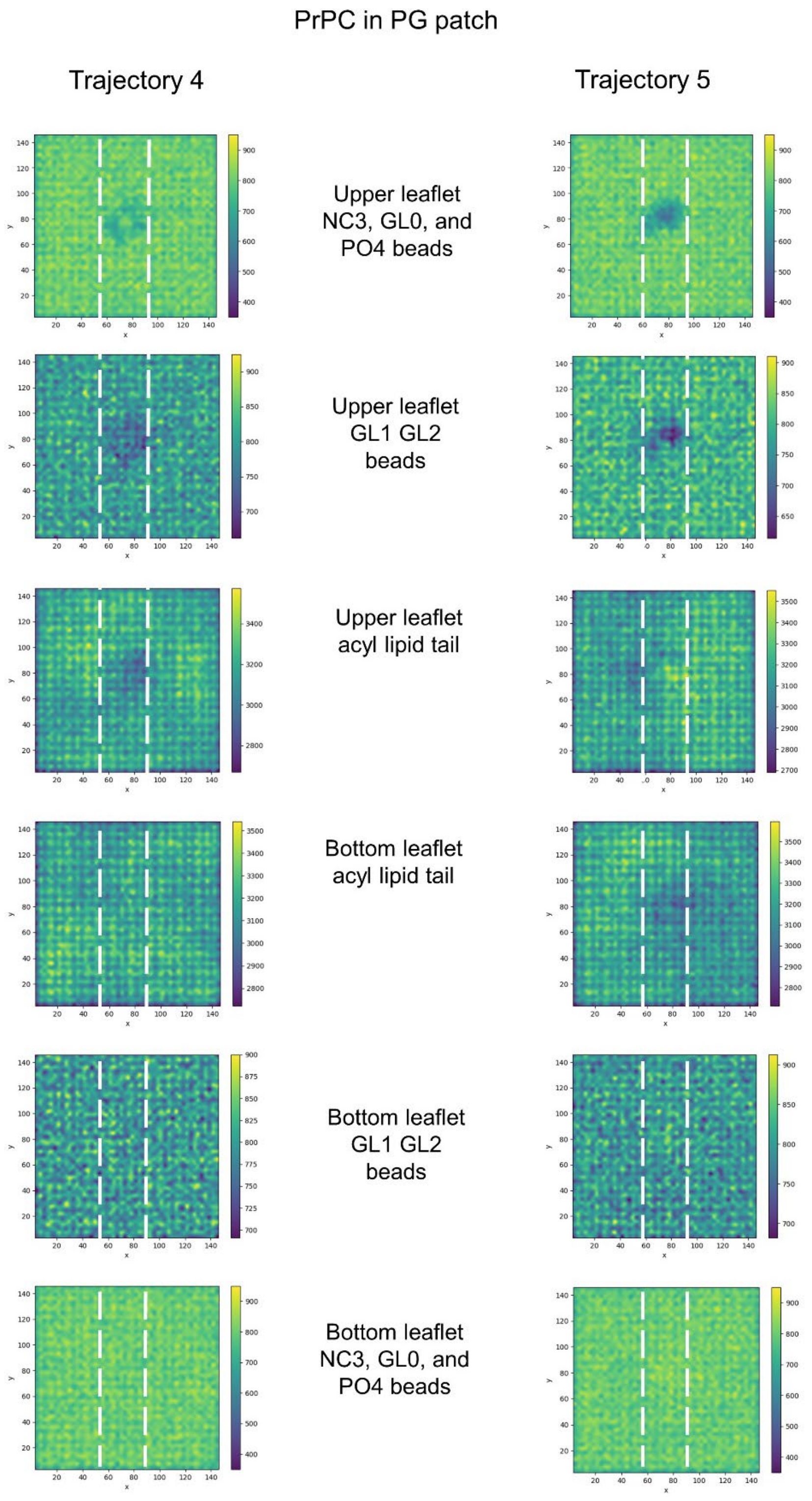

SI Figure 6

2D cumulative histograms only for globular PrP^{C} in PG patch trajectories 4 and 5 showing well-defined divots. Each row corresponds to the 2D cumulative histogram of a set of beads in the upper and bottom leaflets as labeled in the figure. Trajectory 3 did not show a well-defined divot.

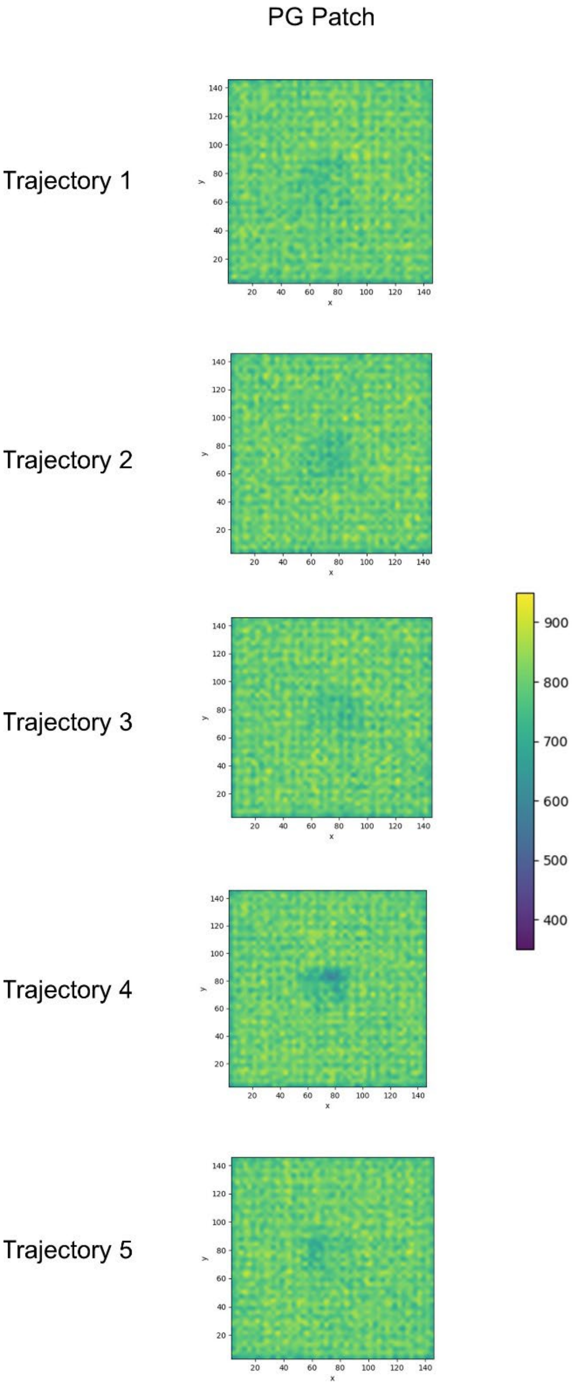

SI Figure 7

2D cumulative histograms of the beads representing the headgroup of the PG lipid for Doppel in PG patch.
